# Fathers’ Involvement: Correlates and Consequences for Child Socioemotional Behavior in the United Kingdom

**DOI:** 10.1177/0192513X15622415

**Published:** 2015-12-14

**Authors:** Anne McMunn, Peter Martin, Yvonne Kelly, Amanda Sacker

**Affiliations:** 1 University College London, London, UK; 2 Anna Freud Centre, London, UK

**Keywords:** child socioemotional behavior, fathers’ involvement, United Kingdom, Millennium Cohort Study

## Abstract

This study investigated longitudinal relationships between fathers’ involvement, as measured by reading, and child socioemotional behavior between infancy and age 7 in 9,238 intact two-parent families from the U.K. Millennium Cohort Study, a national cohort of British children born between 2000 and 2002. Once a variety of covariates and the potential bidirectional nature of relationships were taken into account, a path model showed that fathers’ involvement with their children in infancy significantly predicted better socioemotional behavior at age 3, although the relationship was not strong. Fathers’ reading with their children between ages 3 and 7 was not significantly associated with child socioemotional behavior, but mothers’ reading with their children at age 3 was significantly associated with improved child socioemotional behavior at ages 3 and 5. Results also suggested that parenting in the 21st-century British context remains fairly gendered. Both mothers and fathers were more likely to engage in physical activities with their sons and artistic activities with their daughters. Fathers’ reading was socially patterned in predicted directions.

Interest in the role of fathers has grown as cultural expectations regarding gendered roles within the family began to shift over the latter part of the 20th century (Coltrane, 1996; Doucet, 2013; Goldscheider, Bernhardt, & Lappegard, 2014). One widely used conceptualization of fathers’ involvement is that of Lamb, Pleck, Charnov, and Levine (1987) which theorizes three components of fathers’ involvement: fathers’ engagement, accessibility, and responsibility. *Fathers’ engagement* is defined as direct interaction with the child in the form of caretaking, play, or leisure. *Accessibility* refers to the father making himself available to the child and *responsibility* to the provision of resources for ensuring that the child is taken care of (Lamb et al., 1987; Pleck & Masciadrelli, 2007; Pleck, 2010). Pleck and Masciadrelli (2007) further refined the concept of engagement to focus on *positive engagement* activities, which are considered likely to promote child development, whereas Sayer, Bianchi, and Robinson (2004) distinguished between “routine” child care and more “developmental” teaching and playing activities.

## Fathers’ Involvement: Trends and Correlates

While traditional social norms privileging the breadwinner role in defining fatherhood have waned, there is not currently a great amount of evidence to demonstrate actual behavioral change on the part of fathers. The increased availability of time-use data has been helpful here. In many countries, time-use data have fairly consistently shown that the gender gap in hours spent in domestic labor has reduced, but this is due more to large reductions in the amount of time women spend in domestic labor than to large increases among men (Bianchi, Milkie, Sayer, & Robinson, 2000; Gershuny, 2000; Miranda, 2011; O. Sullivan, 2000). Looking more specifically at child care, Sayer et al. (2004) found the ratio of the amount of fathers’ time to mothers’ time spent in child care increased across all primary child care activities among married couples in the United States between the mid-1960s and the late 1990s. On the other hand, a time-use study in France and the Netherlands found that “new fatherhood” was an image that was not founded on real practice among men in terms of time spent in domestic labor and parenting (Devreux, 2007). Additionally, while fathers may be more involved in their children’s lives than previous generations of fathers, evidence suggests that women remain largely responsible for child care and domestic life (Doucet, 2013).

Levels of fathers’ involvement are not homogenous across society. For example, as gender norms continue to identify men as household providers, and time is a finite resource, we might expect fathers’ involvement with their children to diminish with increasing hours spent in paid work (Hook, 2012). However, evidence suggests that working mothers safeguard time spent with their children, despite increased hours spent in paid work (Bianchi, 2000; Sayer et al., 2004), and a recent U.S. study showed that work hours were not strongly related to fathers’ involvement (McGill, 2014). Within couples, parental time may be conceptualized as a household-level resource. The evidence regarding whether fathers become more involved in parenting to offset longer work hours among their wives or partners is mixed (Zick, Bryant, & Österbacka, 2001), as is that on fathers’ involvement in relation to their own educational attainment or financial resources (Cabrera, Shannon, & Tamis-LeMonda, 2007). Some studies suggest that fathers in professional occupations are able to be more involved with their children due to having more control over their work schedules (Berg, Kalleberg, & Appelbaum, 2003; La Valle, Arthur, Millward, Scott, & Clayden, 2002), but other studies suggest the opposite (Shows & Gerstel, 2009).

## Fathers’ Involvement and Children’s Socioemotional Behavior

Fathers’ increasing involvement may work to strengthen family life, particularly against the current backdrop of busy working parents. Fathers’ involvement may benefit children directly, in terms of increased quantity of parental attention, and indirectly through improvements in mothers’ well-being, (Kiernan, 2005) due to sharing the responsibility of parenting, and/or through more harmonious relationships within the household, which have been linked with more equal sharing (Schober, 2012). Social learning theory would suggest that increased interaction with fathers provides children with an additional opportunity to learn social skills, as well as an additional source of emotional and instrumental support (Leidy, Schofield, & Parke, 2013).

Evidence supports the hypothesis that fathers’ more frequent participation in child-related activities has significant beneficial effects on children’s cognitive, linguistic, and socioemotional development across early childhood independent of mothers’ involvement or household financial resources (Aldous & Mulligan, 2002; Amato & Rivera, 1999; Cabrera et al., 2007; Lang et al., 2014; Shannon, Tamis-LeMonda, London, & Cabrera, 2002; Tamis-LeMonda, Shannon, Cabrera, & Lamb, 2004; Xu, Kushner Benson, Mudrey-Camino, & Steiner, 2010), although associations with child behavioral outcomes are not found as consistently as those with cognitive outcomes (Huerta et al., 2012). Three reviews of the evidence (Downer, Campos, McWayne, & Gartner, 2008; Sarkadi, Kristiansson, Oberklaid, & Bremberg, 2008), including one meta-analysis (Harris, 2010), in relation to fathers’ involvement (defined broadly, ranging from simplistic binary measures of father’s presence or absence to multifactorial constructs) and child socioemotional outcomes have found a significant positive association, although not always for all gender and socioeconomic subgroups. However, there are some gaps in existing evidence; for example, few studies have taken account of mothers’ involvement when investigating fathers’ involvement. Martin, Ryan, and Brooks-Gunn (2007) have shown the importance of investigating the joint influence of mothers and fathers, where appropriate. In addition, relationships between parents’ involvement and child socioemotional behavior may be bidirectional—children’s behavior may influence parents’ involvement as well as parents’ involvement influencing child behavior—and few studies have taken this into account. A small minority of existing studies use longitudinal designs and, to our knowledge, only three examined the potential bidirectionality between fathers’ involvement and child behavior (Downer et al., 2008). In addition, much of the work in this area has been conducted in the United States. Great Britain is fortunate in its relative wealth of birth cohort studies, and currently, three longitudinal studies have investigated the role of fathers’ involvement in child socioemotional development in Britain (Flouri & Buchanan, 2003, 2004; Huerta et al., 2012; Washbrook, 2007), although none examined the potential bidirectionality of relationships between fathers’ involvement and child behavioral outcomes. Three studies have used data from the most recent British birth cohort study, the U.K. Millennium Cohort Study (MCS), which is used here, to examine the relationship between fathers’ involvement and child behavior. Two of these were cross-sectional studies showing a significantly lower likelihood of behavioral difficulties at age 3 (Dex & Ward, 2007), but not at age 5 (A. Sullivan, Cara, Joshi, Ketende, & Obolenskaya, 2010), with increasing fathers’ involvement. More recently, an Organisational for Economic Co-operation and Development study investigated fathers’ involvement and child development longitudinally in the MCS and found that fathers’ involvement was inconsistently associated with lower externalizing scores over time (Huerta et al., 2012).

Parent–child reading has been identified as a form of parental involvement that is particularly important for cognitive development and school readiness (Forget-Dubois et al., 2009; Mistry, Benner, Biesanz, Clark, & Howes, 2010; Raikes et al., 2006; Senechal & LeFevre, 2002) and has been shown to be more strongly related to learning outcomes than parental play or warmth (Davis-Kean, 2005). However, many studies of parental reading focus specifically on mother–child reading (Forget-Dubois et al., 2009; Raikes et al., 2006) and very little is known about father–child reading (Duursma, Pan, & Raikes, 2008). In addition, while parent–child reading has been linked with a variety of measures of school readiness, achievement, and cognitive development, the potential impact of parent–child reading on behavioral development has not been investigated. We hypothesize that the benefits of the child-directed joint-attention episodes that are involved in parent–child reading may extend beyond the development of cognitive and language skills to include the promotion of socioemotional security.

## This Study

The MCS represents an excellent resource for examining longitudinal, bidirectional relationships between fathers’ involvement and child behavior (Twamley, Brunton, Sutcliffe, Hinds, & Thomas, 2013). This article has two aims. First, we examine the extent to which mothers and fathers in this cohort are involved equally with their children and whether the types of activities contemporary British parents do with their children vary by the gender of their child. For this, we examine a variety of aspects of parental involvement, with a specific focus on *engagement* activities as conceptualized by Lamb et al. (1987; see also Pleck & Masciadrelli, 2007; Pleck, 2010). Next, we use data from the first four waves of the MCS to build on previous work by modeling bidirectional relationships longitudinally at four time points when children were between 9 months and 7 years old. Here, we focus on the specific parental activity of reading with children as this is the only measure of fathers’ engagement that is consistently available across time points, so that our bidirectional analysis is by necessity limited to this marker of fathers’ broader involvement. We hypothesize that fathers’ reading with their child will have significant beneficial cross-sectional and longitudinal effects on children’s socioemotional behavior, independent of the effects of mothers’ reading and any effects of previous child behavior on parental reading.

## Method

### Data

MCS is a prospective study of children born in the United Kingdom at the start of the new millennium. The original cohort consisted of 18,819 children born between September 2000 and January 2002, which represented a response rate of 82% of eligible households. In total, there have been five waves of data collection of which the first four, at ages of 9 months, and 3, 5, and 7 years, are used here, with response rates of 78%, 79%, and 72% of the issued sample, respectively. For this analysis, we selected singleton births in “intact” two-parent families in which the same mother and father were present in the household and interviewed at all four waves. Nonresident fathers were not included as different measures of fathers’ involvement are collected for nonresident fathers in the MCS. Defining intact families in this way resulted in a sample of *n* = 9,573, which represents 69% of all responding families in Wave 4, and of these, there were 9,238 children with a valid parent-reported socioemotional behavior score. MCS data are publicly available, and ethical approval for data collection was obtained from a multicenter research ethics committee in Great Britain.

### Measures

#### Father’s and Mother’s Reading

The only measure of father’s engagement with his child that was measured (almost) identically at ages 3, 5, and 7 was father’s reading to child. This question asks: “How often do you read to [child’s name]?” and offers a 6-point response scale (1 = *every day or almost every day*, 2 = *several times a week*, 3 = *once or twice a week*, 4 = *once or twice a month*, 5 = *less often than once a month*, 6 = *not at all*). At age 7, the formulation was changed to “How often do you read *with or* to [child’s name]?” Frequency of reading to child was also measured for mothers in the same way. For descriptive analysis, parental reading is dichotomized as the proportion of parents reading to/with their children several times a week or more and those who read less frequently. In path models testing the bidirectional effects of fathers’ involvement and child socioemotional behavior, parental reading is used as an ordered categorical variable, using all six response categories. Fathers and mothers each report on their own frequency of reading.

#### Early Involvement of the Father

No measure of “reading to child” was available at 9 months. Early father involvement at 9 months was measured through four questions asking the father about the frequency with which he looks after the baby on his own, changes the baby’s nappy, feeds the baby, and gets up for the baby in the night. Each variable was measured on a 6-point response scale ranging from “more than once a day” to “never.” These four variables were combined into a summary score of “Early Father Involvement” ranging from 0 to 20. The resulting scale had moderate reliability, with a Cronbach’s alpha of .68.

#### Other Measures of Father’s and Mother’s Involvement

At ages 5 and 7, the following additional measures of father’s activity with his child were available: telling stories, engaging in musical activities, drawing/painting, physical games (only available at age 5), indoor games, taking to the park or playground, getting the child ready for bed, and looking after the child on his own. All but the last two of these activities were also available for mothers. The response scales were identical to the response scale for frequency of reading described above. As with reading, each parent reports on their own frequency of activity.

#### Child Socioemotional Behavior

Socioemotional behavior was measured by the Total Difficulties scale of the Strengths and Difficulties Questionnaire (SDQ; Goodman, 2001). This 20-item scale measures four components of psychological adjustment in children: Hyperactivity/Inattention, Emotional Symptoms, Conduct Problems, and Peer Problems. A Total Difficulties score was calculated as the sum of the component scores if all component scores were valid. Component scores were prorated if no more than 2 out of 5 items in a subscale had missing values. The SDQ (filled in by the main respondent, usually the biological mother) was available at ages 3, 5, and 7. This parent version of the SDQ, has been shown to correlate highly with both the Child Behavior Checklist and the Rutter questionnaire, and to discriminate between cases of child psychological morbidity and controls at least as well as these instruments (Goodman, 2001). The distribution of all SDQ scores in this sample was positively skewed and leptokurtic, deviating substantially from normality. Nevertheless, use of scale scores as quasicontinuous outcomes in linear regression are justified by the large-sample properties of regression estimates that imply that significance tests on regression coefficients can be valid even with nonnormal outcomes (Lumley, Diehr, Emerson, & Chen, 2002). Transformation of scores did not affect the relationships seen and the original metric was therefore preferred.

#### Covariates

Each of the following covariates were available at each wave and included at 9 months, and ages 3, 5, and 7 in bidirectional path analysis unless otherwise specified.

##### Household characteristics

Household income was measured as a categorical variable comprising six income bands. Household income bands take account of the number of parents in the household, but are not equivalized for the number of children; however, the number of children in the household was also included in multivariate analysis. Information on the employment status of mothers and fathers was combined to create a measure of household employment status with four categories: “Dual Earner,” “Male Breadwinner,” “Female Breadwinner,” and “Both Unemployed.”

##### Child characteristics

Child’s gender was measured at 9 months. Infant temperament at 9 months was measured by 14 questions from the Carey Infant Temperament Scale (Carey & McDevitt, 1978). These items were subjected to an exploratory factor analysis, and a three-factor solution was found to achieve simple structure. Variables uniquely loading onto each factor were then summed to create scale scores of three concepts, corresponding to original subscales of the Carey Infant Temperament Scale: Mood (five items, Cronbach’s alpha = .55), Regularity (four items, Cronbach’s alpha = .71), and Approach Withdrawal and Adaptability (five items, Cronbach’s alpha = .68).

##### Parental characteristics

Fathers’ and mothers’ age were each measured at 9 months, and mean-centered when used in multivariate analysis. Highest educational qualification of each parent was categorized by British National Vocational Qualifications (NVQ) ranging from NVQ5 (equivalent to postgraduate qualifications) to NVQ1 (equivalent to D-G grade on General Certificate of Secondary Education in Britain or some high school education in the United States) and no qualification. Those with “overseas qualifications” were excluded in order that educational qualifications could be treated as an ordinal variable for path modeling. Nonlinear effects were expected for parental work hours, and distributions of mothers’ and fathers’ work hours were markedly different. Therefore, categorical work hours variables were constructed separately for fathers and mothers. Fathers: “not in work,” “part time (under 35 hours per week),” “full time (35 to 40 hours),” “overtime (41 to 59 hours),” and “long hours (60 or more hours per week).” Mothers: “not in work,” “short hours part time (under 20 hours per week),” “part time (20 to 34 hours),” “full-time (35-40 hours),” and “overtime (41 or more hours per week).” Occupational social class for both parents was measured according to the five-category version of the British National Statistics Socio-Economic Classification (Rose & O’Reilly, 1997): “Managerial and Professional,” “Intermediate Occupations,” “Small Employers and Own-Account Workers,” “Lower Supervisory and Technical,” and “Semiroutine and Routine.” Depressive symptoms in mothers and fathers were measured by the six-item Kessler Scale of Psychological Distress at ages 3, 5, and 7. Scores ranged from 0 to 24. For descriptive analyses, parents with 13 or more depressive symptoms were considered to be at risk of depression. This scale was not available at the first wave; instead, mothers’ and fathers’ depression was measured by the 9-item “Malaise” scale (Rutter, Tizard, & Whitmore, 1970). The scale’s reliability in this sample as measured by Cronbach’s alpha was .71.

### Analytic Techniques

Descriptive statistics were employed to compare the frequency with which fathers and mothers engaged in a number of activities with the cohort child. For the purpose of presentation, in the descriptive analysis, activity variables were dichotomized into the categories “at least several times a week” and “less often.”

To estimate bidirectional effects of father’s involvement on child well-being over time, we conducted a path analysis (Stage, Hasani, & Amaury, 2004), using continuous measures of parent-rated SDQ scores and continuous measures of fathers’ and mothers’ reading from Waves 2 to 4. Path analysis is an extension of linear regression, which allows for direct and indirect effects between all of the variables from multiple time points to be estimated simultaneously. This allows us to estimate whether relationships are consistent with our hypothesis that fathers’ involvement significantly predicts better (lower) child behavior scores independent of mother’s reading and any effects of previous child behavior on parental reading. Initial conditions measured at Wave 1 included child temperament and the measure of father involvement with his baby. In addition, we controlled for a set of demographic, parental, and child covariates at all time points. The path analysis was carried out using M*plus* 5.2.

#### Weights

All analyses were conducted using combined survey design and nonresponse weights for the appropriate wave. In statistical models using data from multiple waves, Wave 4 weights were used. The svy command in STATA 12 was used to account for the clustering in the data due to the MCS sampling design.

#### Missing Values

In bivariate analyses, pairwise deletion of missing values was employed. For multivariate analysis, we used multiple imputations to replace missing values of independent variables. No outcome values were imputed. We used the chained equations approach available in STATA 12 (Royston & White, 2011) to define appropriate models for the imputation of continuous, ordinal, and nominal variables. We applied Rubin’s (1987) rules: Missing values were imputed five times, multivariate statistical analyses were then conducted for the five filled-in data sets, and estimates were averaged over the five analyses. Standard errors were adjusted to take into account the additional uncertainty incurred through the need to impute missing values (Schafer & Graham, 2002).

## Results

### Involvement in Parenting Activities: Comparing Mothers and Fathers by Child Gender

Table 1 shows the proportion of fathers and mothers who participate in various activities with their children (several times per week or more) at age 5 and at age 7 separately by the gender of the child. At age 5, fathers were significantly more likely than mothers to play physical games with their children. Otherwise, mothers were significantly more likely than fathers to participate in each of the activities with their 5-year-old children at least several times per week. Reading was the most prevalent activity for both parents, with 81% of mothers and 49% of fathers reading to their 5-year-old at least several times per week. When their children were 7 years old, fathers were more likely than mothers to play games with their child at least several times per week and a little over a fifth of both mothers and fathers told their children stories at least several times per week. On the other hand, mothers were significantly more likely than fathers to read, do artistic activities, and take their child to the playground at least several times per week (Table 1).

**Table 1.** Proportion of Parents Who Participate in Each Activity Several Times a Week or More by Gender of Child.

	Child age 5 (*N* = 8,005)^a^				Child age 7 (*N* = 7,971)^a^			
	Mothers		Fathers		Mothers		Fathers	
	Girls	Boys	Girls	Boys	Girls	Boys	Girls	Boys
Read	82.9	80.9*	49.9	49.9^*ns*^	69.7	70.8^*ns*^	41.8	39.1*
Tell stories	29.1	27.8^*ns*^	24.4	24.7^*ns*^	21.2	20.8^*ns*^	20.0	22.6**
Musical activities	67.1	59.7**	44.4	38.2**	56.4	47.9**	38.8	34.3**
Draw/paint	31.8	23.9**	16.0	13.7**	16.6	11.9**	8.2	7.6^*ns*^
Play physical games	20.7	25.9**	31.5	45.9**	—	—	—	—
Play games/toys indoors	51.0	53.0^*ns*^	43.9	54.9**	27.5	29.9*	30.5	39.4**
Take to park/playground	15.8	17.4*	8.2	10.9**	12.0	14.3**	7.7	10.0**
Get ready for bed	—	—	63.1	67.0**	—	—	61.1	65.5**
Look after on own	—	—	32.4	36.0**	—	—	34.5	36.8^*ns*^

a Sample sizes are unweighted, proportions are weighted.

** *p* < .01. *p < .05 (tests for difference by gender of the child).

Looking at the gender of the child, both mothers and fathers were significantly more likely to participate in artistic activities at least several times per week with daughters than with sons. When children were 5 years old, 32% of mothers of daughters drew or painted at least several times per week compared with 24% of mothers of sons (Table 1). This relationship persisted when children were 7 years old. When cohort members were 5 years old, both mothers and father were significantly more likely to play physical games at least several time per week with sons (46% of fathers and 26% of mothers) than with daughters (32% and 21%, respectively). In addition, fathers of sons were significantly more likely than fathers of daughters to play games, go to the park, put their child to bed, and look after their child on their own at least several times per week (Table 1). Reading and telling stories to or with children did not differ by the gender of the child as greatly as did participation in artistic or physical activities (Table 1).

### Correlates of Fathers’ Reading

Table 2 shows the proportion of fathers who read to their child at least several times per week by fathers’ characteristics. The likelihood of fathers reading to their children at least several times per week increased with father’s educational qualifications, ranging from 28% among fathers who had no educational qualifications to over half of fathers with postgraduate degrees. Fathers aged 30 or older and those in managerial, professional, or intermediate-class occupations were significantly more likely to read frequently than younger fathers or those in other occupational classes. Fathers with lower levels of depressive symptoms were more likely than other fathers to read to their child frequently, but these differences did not reach statistical significance, probably due to the low number of fathers with 13 or more depressive symptoms. There was a curvilinear relationship between fathers’ reading and their work hours so that fathers working 35 to 40 hours per week were the most likely to read to their child frequently, whereas unemployed fathers and those working 60 hours or more per week were the least likely.

**Table 2.** Proportion of Fathers Who Read to Their Child at Least Several Times per Week by Father’s Characteristics, All When the Child Is Age 7.

	%	95% Confidence interval
Father’s educational qualifications (*N* = 8,210)		
NVQ5	51.3	[47.4, 55.3]
NVQ4	48.0	[45.7, 50.3]
NVQ3	39.7	[36.3, 43.0]
NVQ2	33.6	[31.3, 35.8]
NVQ1	32.5	[27.2, 37.7]
No qualifications	28.1	[24.1, 32.0]
Father’s age at birth (years), (*N* = 7,382)		
Below 20	36.5	[14.9, 58.1]
20-24	28.5	[22.6, 34.4]
25-29	37.3	[34.3, 40.4]
30-34	44.7	[42.3, 47.0]
35-39	42.0	[39.5, 44.5]
40 or older	41.1	[37.5, 44.7]
Father’s work hours (*N* = 8,184)		
Not in work	35.4	[31.0, 39.7]
Under 35 hours/week	39.6	[34.7, 44.5]
35-40	42.7	[40.9, 44.5]
41-59	39.8	[37.2, 42.8]
60+	34.6	[30.7, 38.4]
Father’s depressive symptoms (*N* = 7,593)		
Not depressed	41.2	[39.7, 42.7]
At risk of depression	36.9	[29.6, 44.2]
Father’s occupational class (NSSEC), (*N* = 7,455)		
Managerial/professional	46.2	[44.3, 48.1]
Intermediate	46.9	[41.5, 52.3]
Small employer and own account	34.8	[31.6, 37.9]
Lower supervisory and technical	34.1	[30.4, 37.8]
(Semi)routine	34.0	[31.1, 36.8]

*Note*. NVQ = National Vocational Qualifications; NSSEC = National Statistics Socio-Economic Classification.

Table 3 shows the proportion of fathers who read to their child at least several times per week by mothers’ and household characteristics. The likelihood of fathers reading to their children at least several times per week increased with household income and the number of hours mothers worked, ranging from 35% in households in which mothers were not in paid work to 52% among those in which mothers worked more 40 hours per week. Similarly, fathers in dual-earner and female-earner households were more likely to read frequently to their children than fathers in male-breadwinner or no-earner households, and fathers were more likely to read frequently to their child in households in which mothers also read to their child frequently. The proportion of fathers reading frequently to their children decreased as the number of children in the household increased.

**Table 3.** Proportion of Fathers Who Read to Their Child at Least Several Times per Week by Mother’s and Household Characteristics, All When the Child Is Age 7.

	%	95% Confidence interval
Mothers’ characteristics		
Mother’s work hours (*N* = 8,125)		
No work	35.3	[32.9, 37.7]
Under 20 hours/week	41.5	[39.1, 43.9]
20-34	41.7	[39.1, 44.4]
35-40	44.0	[40.4, 47.6]
41+	52.1	[43.5, 60.6]
Mother’s depressive symptoms (*N* = 7,535)		
Not depressed	40.6	[39.1, 42.2]
At risk of depression	35.0	[27.2, 42.9]
Mother’s reading (*N* = 8,151)		
Several times per week or more	44.0	[42.4, 45.7]
Less	31.4	[29.3, 33.5]
Household characteristics		
Household income per week (*N* = 8,211)		
<200	35.0	[28.3, 41.7]
£200-£399	35.6	[32.8, 38.4]
£400-£599	36.5	[34.0, 39.1]
£600-£999	43.1	[40.9, 45.3]
£1,000+	46.7	[43.5, 50.0]
Number of children (*N* = 8,217)		
1	45.5	[41.6, 49.5]
2	43.2	[41.2, 45.2]
3	38.6	[35.9, 41.1]
4+	29.6	[26.8, 32.5]
Household employment (*N* = 8,092)		
Dual earner	42.4	[40.7, 44.1]
Male breadwinner	36.0	[33.3, 38.6]
Female breadwinner	43.9	[36.2, 51.6]
Both unemployed	31.4	[26.2, 36.6]

### Bidirectional Relationships Between Fathers’ Reading and Child Socioemotional Behavior

**Figure 1.**
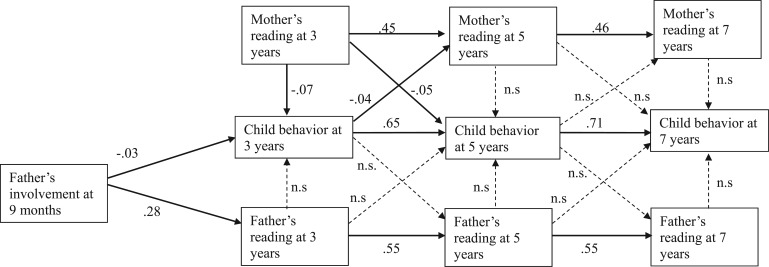
Path model (standardized estimates) of longitudinal relationship between parents’ reading and children’s behavior (*N* = 9,238).^a^ *Note*. RMSEA = root mean square error of approximation; CFI = comparative fit index. Indices of model fit: RMSEA: 0.041; CFI: 0.856. *R*^2^ values for Child Behavior: Age 3: .286; Age 5: .496; Age 7: .551. ^a^Adjusted for 9 months: child temperament, gender, mothers’ and fathers’ age, mothers’ and fathers’ occupational class, mothers’ and fathers’ education, household income, mothers’ and fathers’ working hours, mothers’ and fathers’ malaise. Each concurrent time point: mothers’ reading, mothers’ and fathers’ work hours, mothers’ and fathers’ depressive symptoms, number of siblings.

Figure 1 shows the coefficients from a path analysis of the bidirectional relationship between fathers’ reading and child socioemotional behavior when children were 9 months, 3, 5, and 7 years old. In recognition that father’s involvement in two-parent families is best understood as one part of a mother–father dyad, coefficients for mothers’ reading are also shown. Where relationships were statistically significant at the 5% level or lower, the standardized coefficient is shown, indicating the strength and the direction of the relationship shown by the arrow while simultaneously taking account of each of the other relationships shown in the model as well as adjustments as indicated in the footnotes. The model had good fit according to the root mean square error of approximation (0.041), and a moderate fit according to the comparative fit index (0.856). *R*^2^ values for the three SDQ Total Difficulties scores at ages 3, 5, and 7 were .286, .496, and .551, respectively. Figure 1 shows that father’s involvement at 9 months predicted better socioemotional behavior at age 3, although father’s reading was not significantly associated with child behavior in either direction at any age after accounting for each of the mother’s, father’s, and household characteristics. Fathers’ involvement at 9 months also significantly predicted fathers’ reading at age 3, and parents’ (both mothers and fathers) reading at age 3 significantly predicted parents’ reading at age 5 and reading at age 5 predicted reading at age 7. Father’s involvement at 9 months was not significantly associated with mother’s reading at 3 years (not shown).

Mother’s reading at age 3 was significantly associated with improved socioemotional behavior at both age 3 and age 5 even after accounting for concurrent reading at age 5 and previous socioemotional behavior. Children’s socioemotional behavior at age 3 significantly predicted mother’s reading at age 5; the more behavioral difficulties children had at age 3, the less frequently their mother read with them at age 5. Child socioemotional behavior at age 3 significantly and strongly predicted child socioemotional behavior at age 5, which significantly and strongly predicted behavior at age 7.

The path relationships seen in Figure 1 did not vary substantially by the child’s gender or when the internalizing and externalizing subscales of the SDQ measure were used (results not shown).

## Discussion

This study examined longitudinal, bidirectional relationships between parents’ reading and children’s socioemotional behavior between infancy and age 7. Once a variety of covariates and the potential bidirectional nature of relationships were taken into account, our path model showed no significant association between fathers’ reading and child behavior. However, fathers’ involvement in infancy significantly predicted better socioemotional behavior at age 3, although the relationship was not strong. These results suggest that early father involvement may have a small but significant part to play in the prevention of child socioemotional difficulties, when taken within the broader familial context. Nevertheless, this small contribution may be long-lasting as child socioemotional behavior at ages 3, 5, and 7 is strongly and significantly correlated, so even a relatively small impact in the early years may help set children up on a positive trajectory. There is previous evidence to suggest that father’s involvement with their children in infancy has longer term impacts, predicting improved hormonal reactivity, behavior, and mental health at school age and self-worth in adolescence (Boyce et al., 2006; Grossmann et al., 2002).

Our work suggests that mothers’ reading with children has a greater impact than fathers’ reading on children’s behavior, all else being equal. Frequency of mother’s reading at age 3 was significantly associated with better socioemotional behavior scores for children both cross-sectionally at age 3, and longitudinally at age 5. The greater strength of the relationship between mothers’ reading and child behavior, in comparison with fathers’ reading, may be partly due the beneficial effects of fathers’ reading being drowned out by that of mothers. We have seen that mothers are more likely than fathers to be frequent readers, and that fathers who are frequent readers are more likely to live in households in which mothers are also frequent readers. Also, prior to including mother’s reading in the model, father’s reading at age 3 was significantly associated with children’s behavior at age 3 (coefficient = −0.19, *p* = .021).

This study has also shown that fathers’ involvement in reading with their children is socially patterned in what might be expected directions. Fathers with higher levels of education and income, those in more advantaged occupations and those who are older than 30 years of age are more likely to read with their children frequently. Therefore, children whose fathers read with them are also more likely to live in environments that are more socially and economically advantaged. Previous studies have found fathers’ education to be predictive of fathers’ involvement in other settings (Craig, 2006). Reading was selected as a marker of fathers’ involvement, partly for pragmatic reasons as it was the only such marker to be included at ages 3, 5, and 7 in these data, but reading may be more influenced by parental education and reading skills than other forms of involvement. Indeed, previous work has shown that reading is more strongly associated with fathers’ education and occupational class than other forms of involvement in this data set (McMunn, 2012), and a U.S. study showed that fathers with at least a high school education were more likely to read with their children than those without (Duursma, Pan, & Raikes, 2008).

Time availability also appears to be an important correlate of fathers’ involvement (Sayer et al., 2004). In this study, fathers who worked longer than a 40-hour week were less likely to read with their child frequently, as were fathers in male-breadwinner households and those living with greater numbers of children. It seems that time works as a household resource as well, with fathers in dual-earner households and those where mothers worked longer hours more likely to read frequently with their child. Also using the MCS, Norman (2010) showed that mother’s work hours when the child was 9 months old was a stronger predictor of fathers’ involvement when children were aged 3 years than father’s own work hours when the child was 9 months.

In addition to investigating relationships between parental reading and child behavior, this study examined the involvement of contemporary British fathers with their children in a large, national cohort study of children born at the beginning of the millennium and found that parenting practices in Britain remain fairly gendered in the early 21st century. Unadjusted, descriptive analysis showed that both mothers and fathers were more likely to engage in physical activities with sons and artistic activities with daughters. Child gender seems to have implications for a variety of family processes (Raley & Bianchi, 2006); for example, daughters eliciting more verbal interaction from parents (Leaper & Smith, 2004) and doing more domestic chores (Gager, Cooney, & Call, 1999) than sons. In this study, fathers were a little more likely to be involved with sons than with daughters which is in line with previous work in this same cohort of children (Norman, 2010), although studies in the United States have suggested that fathers’ engagement was not related to the gender of the child (Hofferth, 2003; Sanderson & Sanders Thompson, 2002). Consistent with recent findings in the United States (Lang et al., 2014), this study has also shown that, with the exception of physical play and playing games, mothers continue to participate more frequently than fathers in parenting activities with their 5- and 7-year-olds. This is not surprising given that the United Kingdom is still a long way from achieving a dual-earner/dual-carer model (Gornick & Meyers, 2003), and fathers of young children are still much more likely to work long hours than mothers (Crompton, Brockmann, & Lyonette, 2005).

There are several limitations to this study. First, this study is limited by the availability of consistent measures of fathers’ involvement across the study waves. For this reason we were only able to include parental reading in our bidirectional, longitudinal path model and have not been able to investigate these longitudinal relationships with additional indicators of fathers’ involvement. This limited measure of parental involvement may contribute to the lack of an independent relationship between father’s involvement (as measured by reading) and child behavior in the bidirectional models. Second, this study has examined the frequency of fathers’ engagement with their children, but not the quality of those interactions. We do not know the extent to which father–child interactions were perceived by the child, or the father, to be positive or negative, or whether fathers were engaged in secondary activities (such as using a mobile phone) while participating in the reported activities. These data also do not allow us to look at any level of detail regarding when fathers spend time with their children or for how long. In addition, there is likely to be some measurement error in reports of fathers’ involvement as well as child behavior. In this study, the fact that reports of child behavior are usually reported by the mother, while fathers’ involvement is reported by the father, may help reduce reporting bias in any relationship between the two. A variety of parental and household characteristics that are associated with both fathers’ involvement and child behavior have been included in this study. Nevertheless, there are additional factors that have not been included, some—such as contextual societal and cultural factors that may influence the types and frequency of activities that both parents do with their children—which we were not able to measure in this study, and others—such as the quality of relationships in the home—that are available in this study but warrant further investigation in their own right (Schober, 2012). Finally, this study has been limited to fathers in a traditional context, that is stable, two-parent households. Nonresident fathers and fathers in same-sex relationships have not been included and also warrant further investigation separately. Involvement of nonresident fathers with their children was considered sufficiently different from that of resident fathers to warrant separate, detailed investigation, and, indeed a recent study has examined bidirectional relationships between fathers’ involvement and early child behavior in lone mother families in the MCS (Flouri & Malmberg, 2012).

In conclusion, this is the first study to examine bidirectional longitudinal relationships between fathers’ involvement and child behavior up to age 7 in the U.K. context. From a policy perspective, our analysis suggests that policies and workplace practices that enable fathers to spend time with their children at very early ages may be important for children’s subsequent development. The ongoing imbalance between mothers and fathers in their involvement with their school aged children as documented by this study suggests a continuing need for such policies and practices. While it is important to maintain fathers’ links with paid employment for a variety of reasons, including its positive influence on children’s well-being independent of socioeconomic factors (Hope, Pearce, Whitehead, & Law, 2014; McMunn, Kelly, Cable, & Bartley, 2011), fathers in the United Kingdom remain much less likely than mothers to take their entitled paternity or parental leave or to reduce their hours after the birth of a child (Schober, 2013). After paternity leave entitlement in the United Kingdom was extended to up to 26 weeks in 2011, only 0.6% of eligible fathers took up the additional entitlement (Trade Union Congress, 2013). This lack of uptake may partly be driven by the continued gender pay gap in the United Kingdom, which acts as a disincentive for fathers to reduce their working hours or take parental leave and makes it less likely that fathers will take up the new shared parental leave in April 2015 when British mothers and fathers have equal access to parental leave after the birth of a child (Her Majesty’s Revenue & Customs, 2013). In addition, long work hours and increasing work intensification that characterize the British labor market (Crompton et al., 2005) conflict with an increasing expectation and desire that fathers will be involved with their children (Ranson, 2012). This study suggests the need for a greater focus on supporting fathers in the important contribution they have to make particularly in their children’s earliest years.
